# Genetic Factors and Genotype-Environment Interactions Contribute to Variation in Melanin Production in the Fungal Pathogen *Cryptococcus neoformans*

**DOI:** 10.1038/s41598-018-27813-3

**Published:** 2018-06-29

**Authors:** Himeshi Samarasinghe, David Aceituno-Caicedo, Massimo Cogliati, Kyung J. Kwon-Chung, Volker Rickerts, Aristea Velegraki, Sevim Ackaglar, Jianping Xu

**Affiliations:** 1 Department of Biology, McMaster University, 1280 Main Street West, Hamilton, Ontario, Canada; 2 Department of Biomedical Sciences for Health, Università degli Studi di Milano, Milano, Italy; 3 Molecular Microbiology Section, Laboratory of Clinical Immunology and Microbiology, National Institute of Allergy and Infectious Diseases, NIH, Bethesda, Maryland United States; 4 Robert Koch Institute, Berlin, Germany; 5 Medical School National and Kapodistrian University of Athens, Athens, Greece; 6 Uludag University, School of Medicine, Bursa, Turkey

## Abstract

Cryptococcosis is a common opportunistic fungal infection that often disseminates into the central nervous system, leading to meningitis. Production of melanin pigments during infections is one of the most important virulence factors of its causal agent, the human pathogenic yeast *Cryptococcus neoformans* species complex. However, almost nothing is known about the patterns of variation in melanin production among clinical and environmental strains and the potential effects of such variations on virulence. In this study, we assembled a global collection of *C. neoformans* var. *neoformans* strains and investigated their patterns of melanin variation and potential contributors to such variations. Our analyses revealed that genetic differences and genotype-environment interactions explained up to 59% and 43% of the population’s melanin variance respectively, depending on the tested environments. In comparison, environmental factors alone contributed relatively little to melanin variance. We also identified specific changes within the *LAC1* gene, whose protein product catalyzes melanin synthesis, to be associated with variable melanin levels. This study provides fresh insights into the origin and evolution of virulence traits in fungal pathogens while highlighting the complex interplay between genetic and environmental factors that lead to phenotypic variance.

## Introduction

Melanin pigments, polymers of oxidized substrates with a characteristic black/dark brown color, are ubiquitous in nature with many organisms ranging from microbes to humans producing them for a variety of reasons. In the pathogenic yeast *Cryptococcus neoformans* species complex (CNSC), melanin production is a major virulence factor that promotes the survival of yeast cells inside mammalian hosts^1^. CNSC is an opportunistic environmental pathogen of humans and other mammals capable of causing a range of infections including skin lesions, lung infections and meningoencephalitis, collectively known as cryptococcosis^2–4^. CNSC includes strains of two major serotypes, *C. neoformans* var. *grubii* (serotype A) and *C. neoformans* var. *neoformans* (serotype D), both of which mainly infect immunocompromised individuals^5,6^. Once inside the host, cryptococcal cells are phagocytosed by the macrophages but they can resist being digested and persist as an intracellular parasite^7–9^. Macrophages release bursts of reactive oxygen species (ROS) and reactive nitrogen species (RNS) into the phogosomes to create a highly oxidizing environment that is fatally damaging to most engulfed microorganisms^10^. Previous studies suggest that melanin pigments produced by the cryptococcal cells act as a radical scavenger resulting in the removal of harmful oxygen and nitrogen radicals from the cellular environment^11–14^. Indeed, mutant CNSC strains whose melanin production has been eliminated are avirulent in mice^15,16^.

CNSC has a worldwide distribution and is naturally found in the soil, often in association with pigeon droppings^17^: *C. neoformans* var. *grubii* is the more prevalent variety with strains having been isolated from all continents except Antarctica^18^. *C. neoformans* var. *neoformans* is mostly found in temperate climates with a high prevalence in Northern Europe and the Mediterranean region^19^. The ability to produce melanin pigments likely evolved in CNSC as a defense mechanism against stressors commonly found in its natural habitat, such as UV light and high temperatures^20,21^. The presence of similar stressors inside mammalian hosts may have allowed CNSC to become an opportunistic pathogen^22^.

The melanin biosynthesis pathway in CNSC has been well characterized with all the major steps and substrates identified. Diphenolic compounds such as dopamine are taken up from the environment and shuttled through a series of oxidization reactions catalyzed by a laccase named *LAC1*, which is followed by polymerization to yield the melanin pigments^23,24^. We have observed melanin production to vary between both clinical and environmental strains of CNSC with the yeast colonies ranging from white to increasingly dark shades of brown/black. *LAC1* was shown to be essential for melanin biosynthesis in CNSC through a series of knock-out experiments^16^: however, genetic factors contributing to quantitative variation in pigmentation between natural strains remain largely unknown. A previous quantitative trait loci (QTL) analysis identified five genomic regions that contributed to variation in melanisation among the progeny of a hybrid cross between a serotype A (*C. neoformans* var*. grubii*) strain and a serotype D (*C. neoformans* var*. neoformans*) strain^25^. While one of these QTL regions included *LAC1*, the relationships between specific genetic polymorphisms within *LAC1* and differential pigmentation were not identified. Furthermore, other genes with a potential role in melanin variation in CNSC remain to be elucidated. It is also unclear if and to what extent environmental factors affect the variation in melanin production within CNSC.

In this study, we quantified the melanin production of 54 *C. neoformans* var. *neoformans* (i.e. serotype D, abbreviated CND hereafter) strains originated from 9 countries in 3 continents, in both ideal growth conditions as well as in thermal, oxidative and nitrosative stress. These are three common stressors encountered by cryptococcal cells in their natural habitat as well as inside mammalian hosts during an infection. We also conducted multilocus sequence typing to assess the degree of genetic variation in the study population. Our objective was to assess the contribution of genetic, environmental and genotype-environment interaction factors to variation in melanin production in the study population. We also sequenced the promoter and coding region of *LAC1* in all the strains to identify polymorphisms that were correlated with melanin levels.

## Results

### Variation in melanin production

**Figure 1:**
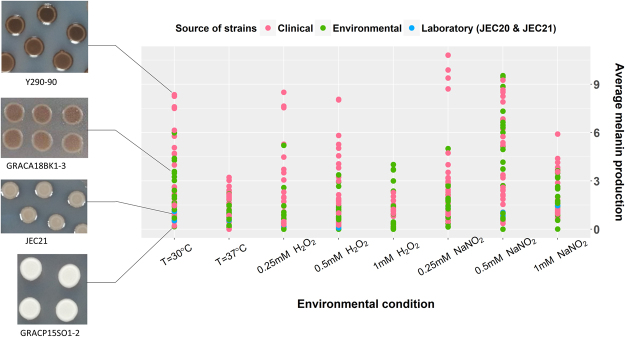
Melanin production of individual C. neoformans var. neoformans strains in different environmental conditions. The melanin production of 54 *C. neoformans var. neoformans* strains was quantified in 8 different conditions: from left, 30 °C, 37 °C, low (0.25 mM H_2_O_2_), intermediate (0.5 mM H_2_O_2_) and high (1 mM H_2_O_2_) oxidative stress, and low (0.25 mM NaNO_2_), intermediate (0.5 mM NaNO_2_) and high (1 mM NaNO_2_) nitrosative stress. Pigment density of the colonies was measured following 3-days’ growth on caffeic acid agar medium. Colonies of a few representative strains from 30 °C are shown on left.

A total of 54 strains of CND from nine countries were analyzed in this study (Table 1). Significant variation in melanin production among these strains was observed at 30 °C after 3-days’ growth, with the highest producer being 52 times more pigmented than the lowest producer of the population (Fig. 1**)**. Similar or slightly higher ranges of melanin levels were observed in low and intermediate oxidative stress, and in low and intermediate nitrosative stress, whereas significantly lower variation among strains was observed in thermal stress, high oxidative stress and high nitrosative stress. Clinical strains (n = 28) produced significantly more melanin on average than the environmental isolates (n = 24) at 37 °C (p = 0.011). In all other conditions, clinical and environmental populations did not significantly differ in average melanin production. Melanin production was found to be correlated with the mating type of the strains in 3 out of the 8 tested conditions. The CND population included 39 *MAT*α strains and 15 *MAT*a strains. In thermal and low oxidative stress, *MAT*α strains produced significantly more melanin than *MAT*a strains (p = 0.0007 and p = 0.03 respectively), whereas in high oxidative stress, *MAT*a strains significantly out-produced *MAT*α strains (p = 0.03). In all other conditions, average melanin production of the *MAT*α strains was higher than that of *MAT*a strains although these differences were not statistically significant. According to ANOVA results, melanin levels were not correlated with geographical origin or source of isolation of the strains.

**Table 1:** Strains of C. neoformans var. neoformans (serotype D) analyzed in this study.

Strain code	Category	Origin	Source	Year isolated	Underlying condition	Molecular Type	Mating type
B3176	ENV	Denmark	Pigeon nest	1970			α
B3179	CLN	Maryland, USA	CSF				α
CAP67-2	CLN	USA					α
GRACA18BK1–3	ENV	Greece	Eucalyptus tree	2013	—	VNIV	a
GRACP15SO1–2	ENV	Greece	Pine tree	2013	—	VNIV	a
GRACP30BK1–1	ENV	Greece	Plane tree	2013		VNIV	a
GRAKI13HO1–1	ENV	Greece	Plane tree	2013		VNIV	a
GRAKI28HO1–1	ENV	Greece	Olive tree	2013		VNIV	a
GRLMM26HO1–2	ENV	Greece	Olive tree	2013	—	VNIV	a
ITMPV22BK7–1	ENV	Italy	Oak tree	2014	—	VNIV	α
IUM 01-0956	SCC	Italy	Skin, head	2001	AIDS	VNIV	α
IUM 01-4729	ENV	Belgium	Pigeon droppings	2001	—	VNIV	α
IUM 01-4730	ENV	Belgium	Dust	2001	—	VNIV	α
IUM 02-0826	VET	Italy	Cat	2002	—	VNIV	a
IUM 02-4295	PPCC	Italy	Skin	2002	No known risk factors	VNIV	a
IUM 77-0033	SCC	Italy	Skin, right ear	1977	No known risk factors	VNIV	α
IUM 79-0801	PCC	Italy	Skin, left leg	1979	Common variable immunodeficiency	VNIV	α
IUM 93-1656	DC	Italy	CSF	1993	AIDS	VNIV	α
IUM 97-4899	PPCC	Belgium	Skin	1997	No risk factors	VNIV	α
IUM 98-2742	SCC	Italy	Skin, left hand	1998	AIDS	VNIV	α
IUM 98-4987	PPCC	Italy	Skin	1998	Breast tumor	VNIV	α
IUM 98-5036	CLN	Belgium	Skin	1998	Diabetes	VNIV	α
JEC20	LAB					VNIV	a
JEC21	LAB					VNIV	α
M9112	PCC	Japan	Skin	1985	No known risk factors	VNIV	α
MAS92-0088	CLN	USA	Blood			VNIV	α
MAS92-0111	CLN	USA				VNIV	α
MAS92-0226	CLN	USA				VNIV	α
MAS92-0232	CLN	USA				VNIV	α
MAS92-0397	CLN	USA				VNIV	α
MAS92-0403	CLN	USA				VNIV	α
MAS92-0621	CLN	USA				VNIV	α
MAS92-0764	CLN	USA				VNIV	α
MAS93-0216	CLN	USA				VNIV	α
MAS94-0176	CLN	USA				VNIV	α
NIH-116	ENV	Virginia, USA	Pigeon nest	1960	—	VNIV	α
NIH-424	ENV	Denmark	Pigeon nest	1970	—	VNIV	α
NIH-426	ENV	Denmark	Pigeon nest	1970	—	VNIV	α
NIH-430	ENV	Denmark	Pigeon nest	1970	—	VNIV	a
NIH-431	ENV	Denmark	Pigeon nest	1970	—	VNIV	α
NIH-432	ENV	Denmark	Pigeon nest	1970	—	VNIV	α
NIH-433	ENV	Denmark	Pigeon nest	1970	—	VNIV	a
NIH-434	ENV	Denmark	Pigeon nest	1970	—	VNIV	α
NIH-442	ENV	Denmark	Pigeon nest	1970	—	VNIV	α
PD1596	VET	Italy	Cat	2010	—	VNIV	α
PD32	VET	Italy	Cat	2009	—	VNIV	α
RKI 13-0490	ENV	Germany	Pigeon droppings	2013	—	VNIV	a
RKI 13-0491	ENV	Germany	Pigeon droppings	2013	—	VNIV	a
RKI 13-0492	ENV	Germany	Pigeon droppings	2013	—	VNIV	a
RKI 14-1061.4	ENV	Germany					a
TRNCGB1H01-1	ENV	Turkey	Pine tree	2013	—	VNIV	α
Y286-90	CLN	Canada	CSF			VNIV	α
Y290-90	CLN	Canada	CSF			VNIV	α
Y494-91	CLN	Atlanta, USA				VNIV	α

PCC = primary cutaneous cyrptococcosis; PPCC = probable primary cutaneous cryptococcosis; SCC = secondary cutaneous cryptococcosis; DC = disseminated cryptococcosis; ENV = environment; VET = veterinary; CLN = clinical; CSF = cerebrospinal fluid; LAB = laboratory.

**Figure 2:**
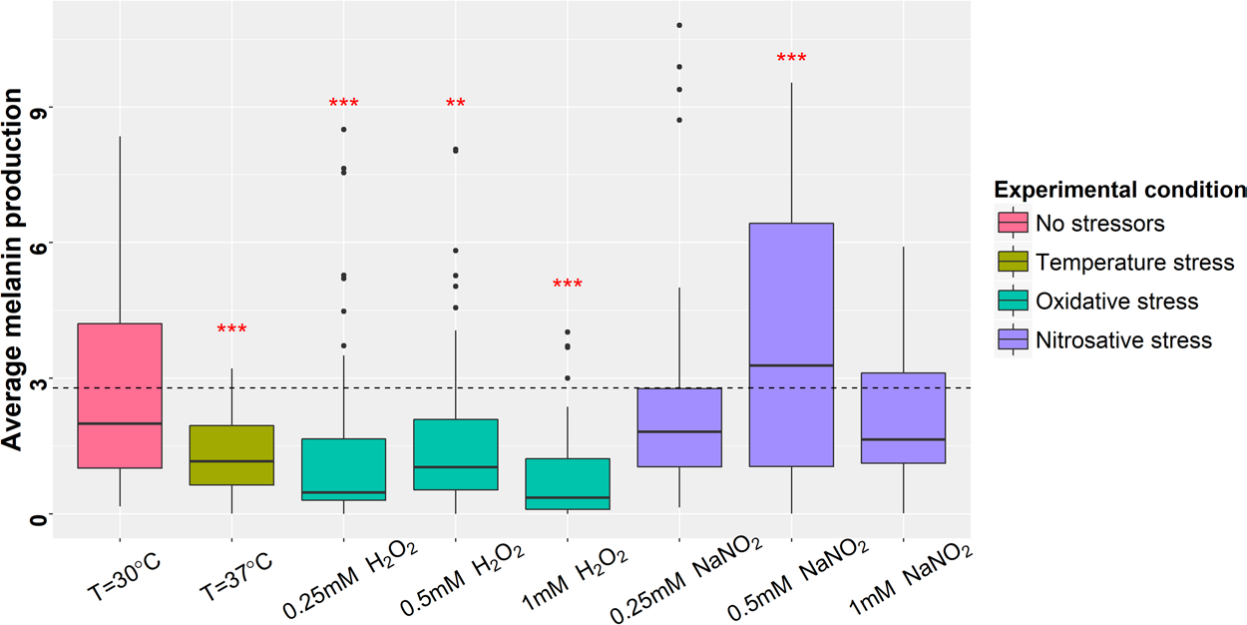
Average melanin production of the C. neoformans var. neoformans population in different environments. The melanin levels of the *C. neoformans* var. *neoformans* population was quantified: from left, in 30 °C, 37 °C, low oxidative stress (0.25 mM H_2_O_2_), intermediate oxidative stress (0.5 mM H_2_O_2_), high oxidative stress (1 mM H_2_O_2_), low nitrosative stress (0.25 mM NaNO_2_), intermediate nitrosative stress (0.5 mM NaNO_2_) and high nitrosative stress (1 mM NaNO_2_). The dashed line represents the average melanin production of the population in the negative control (no added stressors). The red stars above the boxplots represent the degree of statistical significance to which the average melanin production differs from that of the negative control.

**Figure 3:**
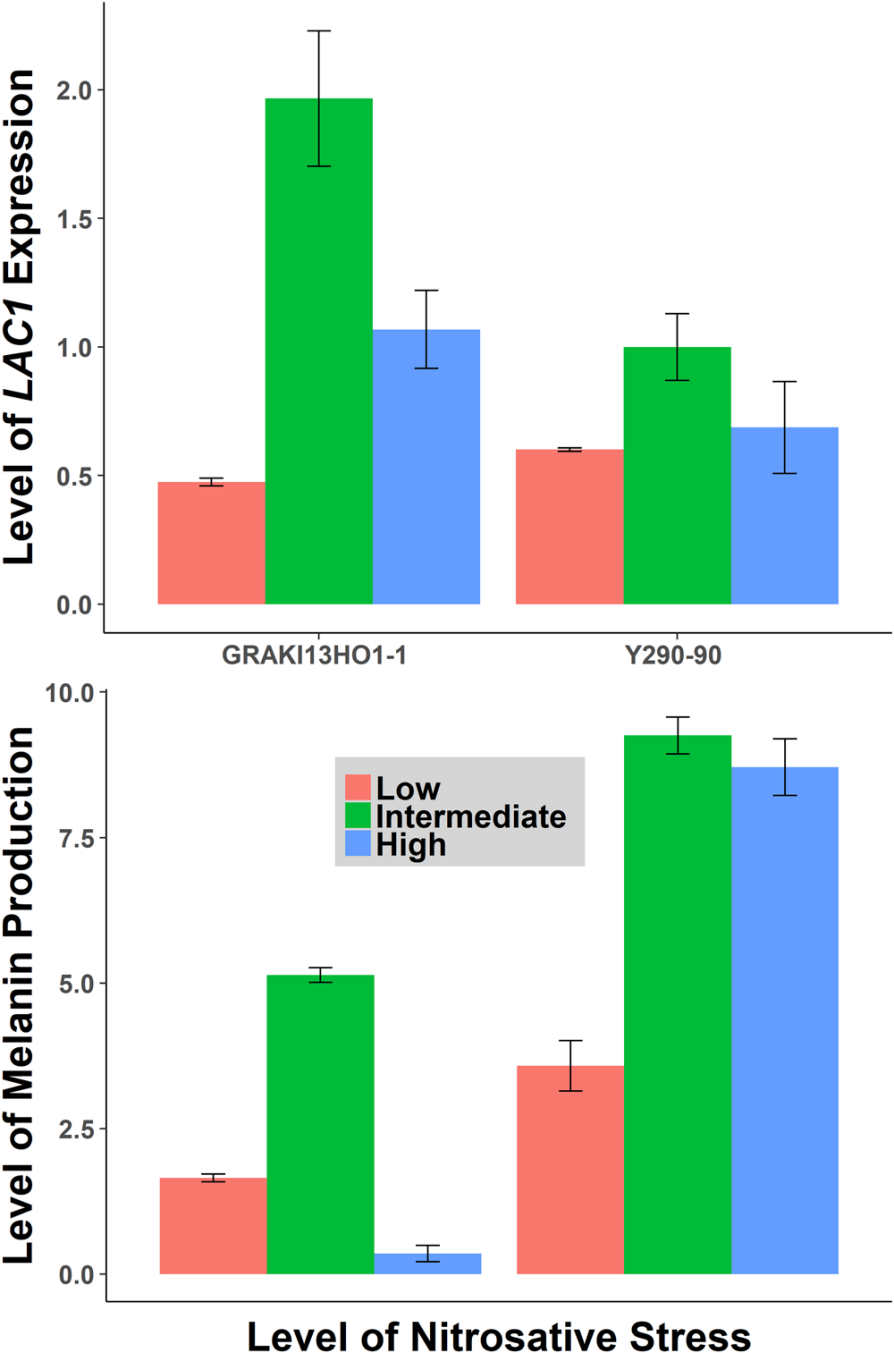
Normalized LAC1 expression in low, intermediate and high nitrosative stress conditions. The abundance of *LAC1* mRNA transcripts was quantified in two representative strains from the study population using RT-qPCR. In both strains, the normalized *LAC1* expression was correlated with the corresponding level of melanin production. *LAC1* expression was induced in intermediate nitrosative stress in comparison to low and high nitrosative stress.

As shown in Fig. 2, the average melanin production of the CND population was significantly higher at the ideal growth temperature of 30 °C with no added stressors than that at thermal stress (p = 1.56E-06) and at all three tested concentrations of oxidative stress (p = 3.09E-05, 0.00459, 5.28E-11 respectively for low, intermediate and high oxidative stress). Interestingly, while the average melanin production was lower in both low and high nitrosative stress (not statistically significant), this was not the case in intermediate nitrosative stress where the average melanin production was in fact considerably higher than that at 30 °C (p = 0.00032). Melanin production in intermediate nitrosative stress was significantly higher than that in low (p = 3.09E-05) and high nitrosative stress (p = 5.75E-11). By quantifying the amount of *LAC1* mRNA transcripts in two representative strains, we found that *LAC1* gene expression was significantly up-regulated in intermediate nitrosative stress compared to low and high nitrosative stress (Fig. 3). Our results suggest that *LAC1* expression and consequently melanin production is upregulated in CND within a narrow window of nitrogen radical concentrations (0.25 mM NaNO_2_ <X>1 mM NaNO_2_).

### Multilocus sequence typing

We performed multilocus sequence typing (MLST) on all 54 CND strains according to the ISHAM consensus scheme for CNSC^26^ which includes seven MLST loci, namely *CAP59*, *GPD1*, *IGS1*, *LAC1*, *PLB1*, *SOD1* and *URA5*. The seven loci are located on six different chromosomes and were shown to be capable of capturing the major genetic variation among CNSC strains^26^. The concatenated sequences of the seven loci resulted in 4092-bp long sequences, alignment of which revealed the presence of 39 different sequence types – STs (Table 2). ANOVA results failed to show any correlation between MLST sequence types and melanin levels in any of the eight tested environmental conditions. Similarly, according to the maximum parsimony trees created (data not shown), STs were not significantly correlated with geographical origin, or source of isolation of the strains.

**Table 2:** MLST allele types and sequence types of the *C. neoformans* var. *neoformans* strains at seven loci. Accession numbers for the full length *LAC1* sequences are also listed here.

Strain	Combined MLST Sequence Type	*CAP59*	*GPD1*	*IGS1*	*LAC1*	*PLB1*	*SOD1*	*URA5*	GenBank Accession of *full LAC1 sequence*
B3176	515	17	21	28	13	13	17	16	MG963844
B3179	160	16	21	30	19	13	17	19	MG963845
CAP67-2	NST4	17	21	28	13	14	17	16	MG963846
GRACA18BK1-3	502	26	38	30	22	13	19	18	MG963863
GRACP15SO1–2	499	26	21	30	22	13	19	18	MG963862
GRACP30BK1-1	498	26	20	30	17	13	19	18	MG963861
GRAKI13HO1-1	503	26	39	30	22	13	19	18	MG963842
GRAKI28HO1-1	500	26	22	44	17	14	23	23	MG963843
GRLMM26HO1-2	489	1	1	1	1	1	27	1	MG963864
ITMPV22BK7-1	496	16	3	30	19	13	17	19	MG963877
IUM 01-0956	521	27	13	12	6	9	8	13	MG963867
IUM 01-4729	510	16	21	24	18	13	20	32	MG963865
IUM 01-4730	514	16	40	24	20	13	20	32	MG963866
IUM 02-0826	517	22	21	30	24	21	17	20	MG963868
IUM 02-4295	516	22	3	31	24	14	17	20	MG963869
IUM 77-0033	135	27	22	43	24	13	17	20	MG963871
IUM 79-0801	520	27	3	43	24	13	17	20	MG963870
IUM 93-1656	279	22	22	31	22	14	17	34	MG963874
IUM 97-4899	507	16	3	26	39	14	20	34	MG963876
IUM 98-2742	112	16	22	31	24	14	17	16	MG963873
IUM 98-4987	518	26	21	31	15	21	19	15	MG963875
IUM 98-5036	506	16	3	24	20	13	20	32	MG963872
JEC20	NST6	17	21	28	19	14	17	20	MG963878
JEC21	NST6	17	21	28	19	14	17	20	MG963879
M9112	168	22	21	30	22	14	17	18	MG963880
MAS92-0088	114	16	21	26	24	18	17	22	MG963852
MAS92-0111	NST3	24	21	84	21	14	17	34	MG963853
MAS92-0226	160	16	21	30	19	13	17	19	MG963855
MAS92-0232	160	16	21	30	19	13	17	19	MG963856
MAS92-0397	114	16	21	26	24	18	17	22	MG963847
MAS92-0403	114	16	21	26	24	18	17	22	MG963848
MAS92-0621	160	16	21	30	19	13	17	19	MG963849
MAS92-0764	165	16	22	48	24	13	23	17	MG963850
MAS93-0216	165	16	22	48	24	13	23	17	MG963851
MAS94-0176	160	16	21	30	19	13	17	19	MG963854
NIH-116	135	27	22	43	24	13	17	20	MG963881
NIH-424	180	20	21	26	21	19	17	21	MG963882
NIH-426	270	16	28	53	24	14	17	20	MG963883
NIH-430	509	16	20	24	16	14	20	16	MG963884
NIH-431	180	20	21	26	21	19	17	21	MG963885
NIH-432	NST1	16	29	24	20	13	20	18	MG963886
NIH-433	515	17	21	28	13	14	17	16	MG963887
NIH-434	NST2	16	21	31	19	13	19	16	MG963888
NIH-442	180	20	21	26	21	19	17	21	MG963889
PD1596	252	22	22	30	19	14	23	41	MG963890
PD32	294	16	21	24	20	13	22	32	MG963891
RKI 13-0490	522	27	21	53	19	14	19	16	MG963858
RKI 13-0491	523	29	21	83	21	14	58	53	MG963859
RKI 13-0492	524	29	21	83	40	14	58	53	MG963860
RKI 14-1061.4	135	27	22	43	24	13	17	20	MG963857
TRNCGB1H01-1	497	16	21	24	20	13	1	32	MG963892
Y286-91	160	16	21	30	19	13	17	19	MG963893
Y290-91	160	16	21	30	19	13	17	19	MG963894
Y494-91	NST6	26	21	32	19	13	22	20	MG963895

NST = New Sequence Type.

### *LAC1* gene sequence

**Figure 4:**
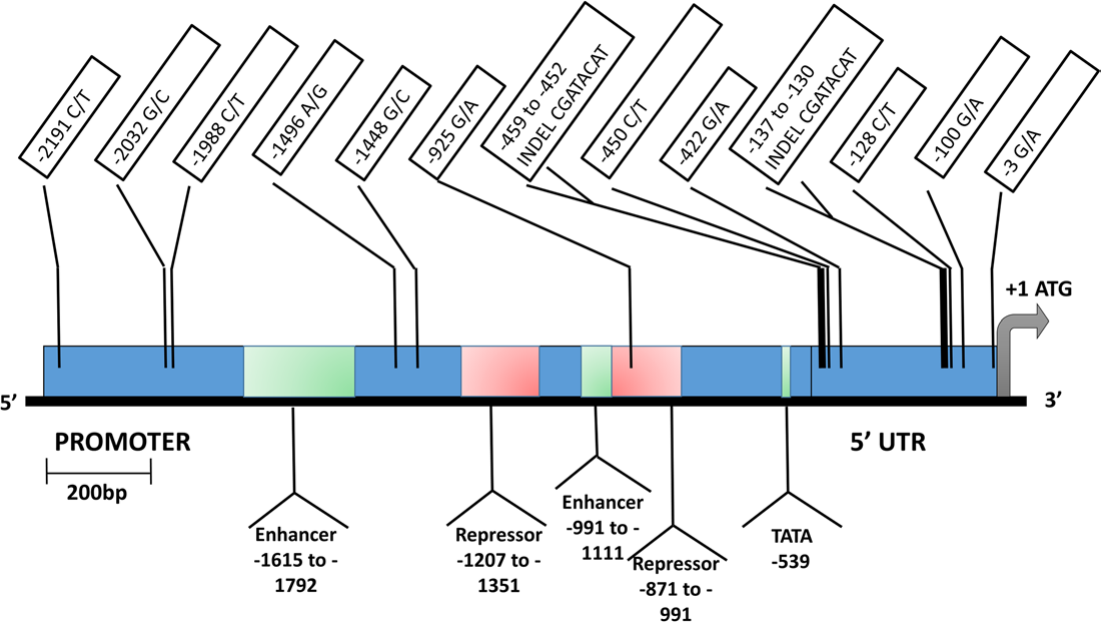
LAC1 promoter and 5′ UTR. This schematic shows the Single Nucleotide Polymorphisms (SNPs) and INDELs that we found within the promoter and 5′ untranslated regions (UTR) of the *LAC1* gene, in our study population of *C. neoformans* var. *neoformans*. Only those polymorphisms with a minor allele frequency threshold ≥10% are shown here. The green regions within the promoter represent transcriptional enhancer elements and the red regions represent the transcriptional repressor elements identified in a previous study^27^. One SNP, −925 G/A, was located within a transcriptional repressor element.

**Figure 5:**
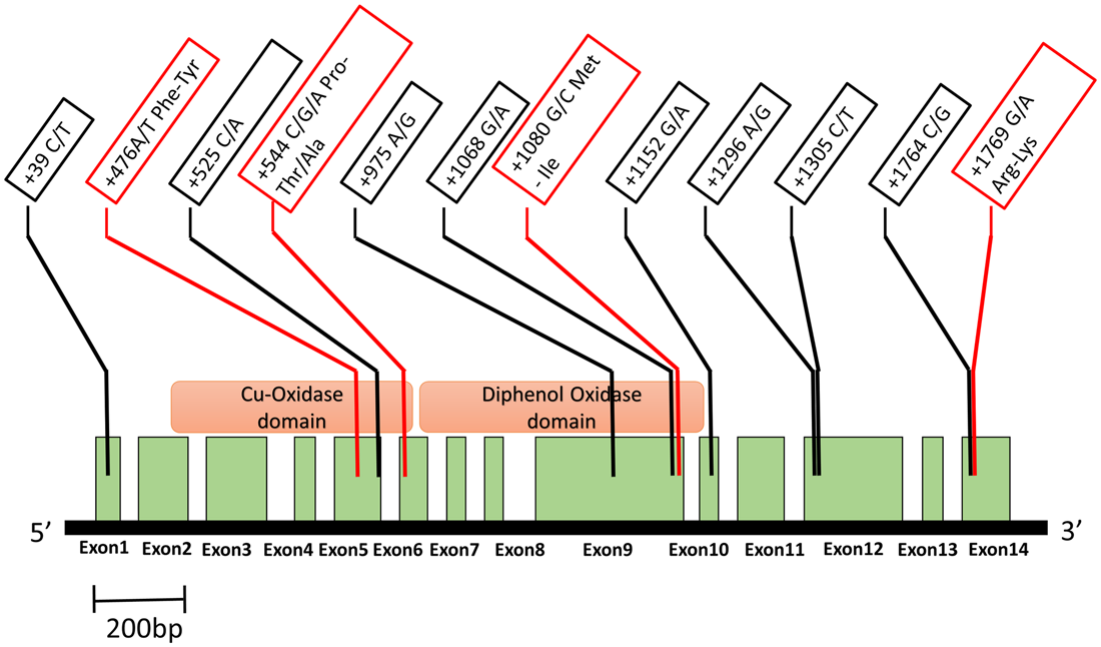
LAC1 coding region. This schematic shows the Single Nucleotide Polymorphisms (SNPs) that we found within the coding region of the *LAC1* gene, in our study population of *C. neoformans* var. *neoformans*. Only those SNPs with a minor allele frequency ≥10% are shown here. The green boxes represent exons and the white boxes in between represent the introns. The four SNPs highlighted in red cause non-synonymous amino acid changes whereas the rest are synonymous SNPs. Three of the four non-synonymous SNPs are located within putative catalytic domains of the Lac1 protein.

The *LAC1* gene fragment sequenced as part of MLST typing was 473-bp in length and most of it was located within an intron. To further elucidate genetic polymorphisms within *LAC1*, we sequenced the entire *LAC1* gene and its promoter in all 54 strains adding up to a total length of 4835-bp. A total of 110 single nucleotide polymorphisms (SNPs) were found within the combined promoter, exons, and intronic regions of the *LAC1* gene, leading to 21 different *LAC1* alleles in the population. Fifteen SNPs were present at a minor allele frequency (MAF) ≥10%, with 11 of them located in the promoter region (Fig. 4) and 4 SNPs causing non-synonymous amino acid changes in the coding region (Fig. 5). Concatenating these 15 SNPs identified the presence of 11 different *LAC1* haplotypes with the majority of the strains (42/54) belonging to 4 haplotype groups. The haplotypes were numbered from 1 to 11 depending on their prevalence with 1 being the most prevalent (14/54) and 11 being the least common (1/54). Strains of similar origin (clinical vs. environmental) tended to cluster within haplotypes. For example, in haplotype 1, 12 out of the 14 strains were environmental, whereas in haplotype 2, 8 out of the 9 strains were of clinical origin.

Our analyses identified that *LAC1* haplotypes were significantly associated with melanin production in thermal stress (p = 0.0138) and high oxidative stress (p = 0.00602). Within these two conditions, we tested the 15 SNPs individually using ANOVA tests to identify those that were significantly associated with melanin production. One SNP in the promoter region, A-1496G, was significantly correlated with melanin production in thermal stress (p = 2.86E-03). Strains with G at this position produced significantly more melanin than strains with A at 37 °C. In high oxidative stress, two SNPs, A-925G and C544G/A, were significantly correlated with melanin production. The former is located within a previously identified repressor region of the *LAC1* promoter^27^ whereas the latter is located within the putative copper-oxidase domain of the LAC1 protein and causes a non-synonymous amino acid switch from proline to threonine or alanine (P182T/A). Eight out of the 54 strains (GRAKI13HO1-1, GRAKI28HO1-1, GRACA18BK1-3, GRACP15SO1-2, GRACP30BK1-1, GRAKI13HO1-1, GRAKI28HO1-1, GRLMM26HO1-2, IUM 01-0956 and M9112) contain alanine at this position and produce significantly more melanin than the rest of the population.

### Contribution of genetic, environmental and genetic-environmental interaction factors to melanin variation

**Figure 6:**
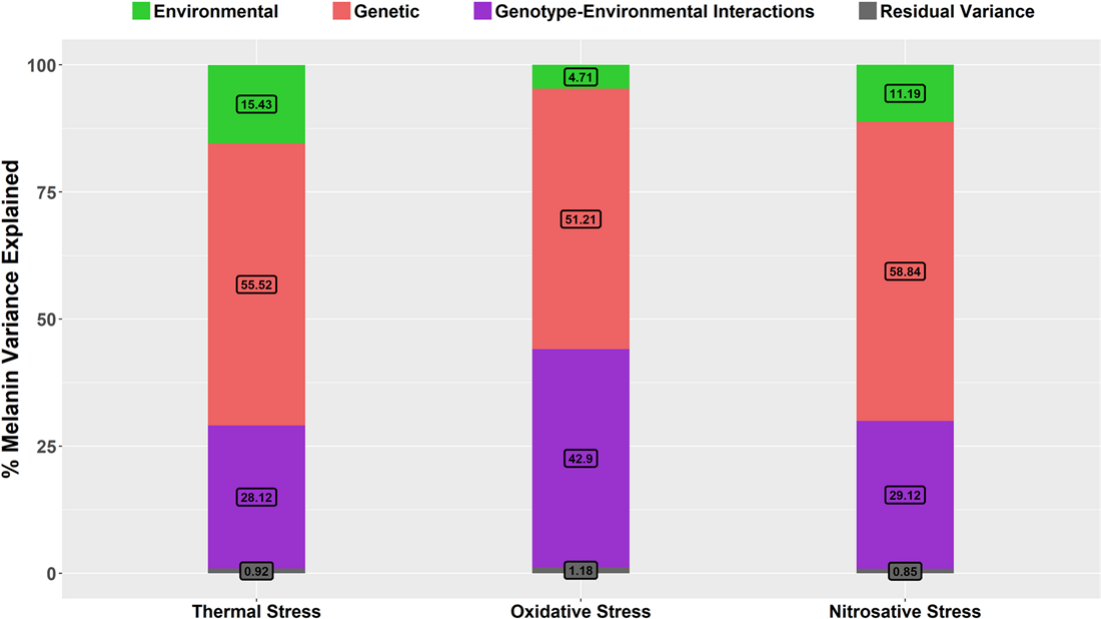
Percent Contribution of genetic factors, environmental factors and genotype-environment interactions to melanin variance. Results of two-way ANOVAs revealed the % variance of melanin production within thermal, oxidative and nitrosative stress environments explained by genetic differences between strains, environmental factors and genotype-environment interactions.

We conducted a series of two-factor ANOVAs to determine the relative proportion of melanin variance explained by overall genetic differences, environmental factors and genotype-environment interactions, as well as the *LAC1* gene and the *MAT* locus. The combined genetic factors were the biggest contributor to the overall variation in melanin production in all three stress conditions with them explaining 56%, 51% and 59% of the variance in thermal, oxidative and nitrosative stresses respectively (Fig. 6**)**. Genotype-environment interaction effects were also a significant contributor at 28%, 43% and 29% respectively. In contrast, though statistically significant, environmental factors alone explained relatively little of the overall melanin variance at 15%, 5% and 11% respectively. Among the genetic factors, the *LAC1* locus accounted for 13% to 30% of the total variance depending on the environmental condition. In thermal and oxidative stress, the contribution of *LAC1* to melanin variance increased with higher levels of stress. However, in nitrosative stress, the biggest contribution from *LAC1* to melanin production was found at intermediate nitrosative stress. Specifically, the *LAC1* gene explained 18% of the total melanin variance at 30 °C, 30% at 37 °C, 13% in low oxidative stress, 21% in intermediate oxidative stress, 23% in high oxidative stress, 23% in low nitrosative stress, 27% in intermediate nitrosative stress, and 22% in high nitrosative stress (Table 3). The contributions of the *MAT* locus to melanin variation were assessed for all three environmental conditions in which it was significantly correlated with melanin levels. Specifically, *MAT* explained 11% of the total melanin variance in thermal stress, 4.4% in low oxidative stress and 4.8% in high oxidative stress.

**Table 3:** Percent melanin variance explained by tested factors.

Tested factors	% Melanin variance explained by tested factors in different stress conditions							
	Thermal stress		Oxidative stress			Nitrosative stress		
	30 °C	37 °C	Low	Intermediate	High	Low	Intermediate	High
Genetic factors	55.52		51.21			58.84		
*LAC1*	17.76	29.83	13.08	21.12	23.17	22.85	26.74	22.12
*MAT*		29.83	4.43	4.85				
Environmental factors	15.43		4.71			11.19		
Genotype-environment interactions	28.12		42.9			29.12		

The % melanin variance explained by genetic factors, environmental factors, genotype-environment interactions, *LAC1* gene and the mating type-determining locus (*MAT*) was determined through two-factor and one-factor ANOVAs, and are summarized in this table. In all three types of stress, genetic factors explained the most variance followed by genotype-environment interactions and environmental factors.

## Discussion

Melanin production is a major virulence factor of the CNSC that contributes to its pathogenicity. While it has been shown that eliminating melanin synthesis makes CNSC strains avirulent, little is known about quantitative variation in melanin production that exists within natural populations of CNSC. Factors contributing to this phenotypic variance remain largely unexplored and elucidating these factors will shed light on evolution and regulation of melanin biosynthesis in CNSC. Our results showed that extensive variation in level of melanisation exists between strains of *C. neoformans* var. *neoformans*. In addition, both genetic factors as well as genotype-environment interactions explain a significant degree of this observed variance in melanin production.

The previous QTL study identified five genomic regions that together explained 49% of the melanin variance observed among progeny strains derived from a hybrid cross between strains of *C. neoformans* var. *grubii* and *C. neoformans* var. *neoformans*^25^. Apart from *LAC1*, the MLST loci sequenced in our study were not located within or near these genomic regions. While *LAC1* was one of the markers identified in the QTL analysis, the MLST sequence only included a 473-bp fragment covering mostly an intronic region of the gene. Therefore, the lack of association between MLST genotypes and melanin levels in our study is not unexpected. While *CAP59*, *GPD1*, *IGS1*, *PLB1*, *SOD1* and *URA5* genes perform important functions within the cryptococcal cell, our results suggest that polymorphisms at these loci do not contribute to variation in melanin production among CND strains.

The previous QTL study determined that *LAC1* explained ~10% of the melanin variance observed at 30 °C, which is lower than our estimate of 18%. This discrepancy could be attributed to a couple of factors. First, due to their historical divergence^28^, there might be more loci involved in melanin variation between the two varieties than within CND, diminishing the contribution of *LAC1* to the overall variance in the hybrid cross. Second, while there are only two alleles at the *LAC1* locus in the hybrid progeny population, a total of 21 alleles were found in our CND sample of 54 isolates. This higher allelic variation at the *LAC1* locus could explain the higher contribution of *LAC1* to the total melanin variation among our study population compared to the lab-derived progeny population from a hybrid cross of the previous study. Regardless, we would like to emphasize that the three *LAC1* SNPs identified here as being associated with melanin variations were only statistically suggested correlations on how genetic polymorphisms at *LAC1* might be related to phenotypic diversity. Targeted (i.e. site-directed) mutagenesis and/or allele-swap experiments are needed in order to determine the causal effects of individual SNPs or alleles on melanin production in different environmental conditions.

Our analyses identified genotype-environment interactions as a significant contributor to melanin variance between strains of *C. neoformans* var. *neoformans*. Dual-use virulence hypothesis proposes that the virulence factors of CNSC, including melanin biosynthesis, evolved primarily as a response to environmental stress, and not to host conditions^22^. If true, this suggests that strains originating from different climatic zones should have differential expression of the virulence factors consistent with the environmental conditions to which they were exposed in their natural habitats. Our findings are consistent with this hypothesis, as the presence of genotype-environment interactions indicate the evolution of genotypes best suited to their environments of origin. This is further highlighted by the increasingly higher contribution of *LAC1* to melanin variance in higher levels of thermal and oxidative stress which points to a complex interaction between *LAC1* and environmental stress.

Melanised cryptococcal cells have shown to be more resistant to temperature extremes and oxidative stress^21,29^. However, in our study population, melanin production was significantly repressed in both thermal and oxidative stress after 3-days’ growth when melanin measurements were taken. Laccase activity was previously found to be severely inhibited at 37 °C^30^, which is consistent with melanin production being repressed at this temperature. We observed that strains gradually became more pigmented over the next several days. This result suggests that melanin production may not be an important component of cryptococcal cells’ initial response to thermal stress or oxidative stress encountered during the early stages of an infection, but it likely plays an important role later on. Taken together, our results suggest that melanin pigments may contribute more to the persistence of a cryptococcal infection, rather than to its initial establishment.

Nitrosative stress caused by RNS is believed to act in a very similar manner to oxidative stress in exerting its microbicidal effects^31^. However, our findings suggest that melanin biosynthesis in *C. neoformans* var. *neoformans* is regulated differently in response to nitrosative stress as compared to oxidative stress. Melanin production seems to be induced in the presence of a moderate concentration of RNS in the environment. A potential mechanism that mediates this response can be found in an *in vitro* study conducted by Matuszak and colleagues where they reported that the concentration of synthetic DOPA-melanin pigments in an acidic buffer increased four-fold in the presence of 0.5 mM NaNO_2_ whereas the presence of H_2_O_2_ in the medium did not produce the same effect^32^. They proposed that the interaction of DOPA-melanin with nitrite at acidic pH leads to melanin oxidation, followed by the generation of a large number of melanin radicals. To the best of our knowledge, our finding is the first report of an *in vivo* system where an intermediate level of NaNO_2_ (0.5 mM) upregulated melanin biosynthesis. Furthermore, we found that this fine-tuned melanin response to RNS was mediated through differential *LAC1* expression. How nitrosative stress levels are perceived by cryptococcal cells to regulate melanin biosynthesis remains to be examined.

In conclusion, our findings indicate the presence of extensive intra-varietal variation in melanisation in the *C. neoformans* species complex, while highlighting the contribution of genetic factors, as well as genotype-environment interactions to the observed variance. Our results indicate that melanin production in CNSC is influenced by multiple factors and its biosynthesis is sensitive to and highly regulated by different environmental factors. Further research is needed to fully elucidate the identity of these factors and how they shape melanin production in response to stressors in its natural as well as host environments.

## Materials and Methods

### Strains

A total of 54 strains of *Cryptococcus neoformans* var. *neoformans* (serotype D) were analyzed in this study, including two lab strains (JEC20 and JEC21) and 52 natural strains (Table 1). Among these 52 natural strains, 14 were from the United States, 11 from Italy, 9 from Denmark, 6 from Greece, 4 from Belgium, 4 from Germany, 2 from Canada, 1 from Japan and 1 from Turkey. Twenty-four of the natural strains were isolated from the environment, 25 from human cryptococcal infections and 3 from veterinary sources. We confirm all 25 clinical strains analyzed here were obtained following the respective institutional and national guidelines where informed consent was given to individual patients and that most of these strains have already been reported in previous publications^19,28,33^. Among the 52 natural strains, 15 strains were of the mating type ‘a’ (*MATa*) and 37 were of mating type ‘alpha’ (*MATα*). The common laboratory strains JEC20 (*MATa*) and JEC21 (*MATα*), both of serotype D, were included in the population as reference strains.

### Melanin quantification

Melanin production was quantified using a spot densitometry assay on caffeic acid-containing solid agar medium as described previously^25^. Caffeic acid is a structural analog of L-DOPA and previous studies have shown that *C. neoformans* uses these two substrates interchangeably to synthesize melanin^24,34^. Briefly, for our assay, the stock cultures maintained at −80 °C were first revived on solid YEPD medium and grown for 2–3 days at 30 °C. Yeast cells from freshly grown cultures were suspended in sterile water and the cell density was adjusted to 1 × 10^6^ cells/ml using the hemocytometer cell counter. A 5 µl drop of this solution was spotted onto the caffeic-acid agar^35^. The plates containing the spots were then incubated in one of eight different environmental conditions for 72 hours, after which measurements were taken. As the negative control, melanin production was quantified at 30 °C, the optimum growth temperature of *C. neoformans*, with no added stressors in the medium. To create thermal stress, plates were incubated at 37 °C. To create oxidative stress, hydrogen peroxide (H_2_O_2_) was added to the caffeic acid agar in one of three concentrations (0.25 mM, 0.5 mM and 1 mM). To create nitrosative stress, sodium nitrite salt (NaNO_2_) was added to the caffeic acid agar in one of three concentrations (0.25 mM, 0.5 mM and 1 mM). Following the 72-hour incubation period, a transilluminator was used to expose the resulting colonies to reflective white light, and the amount of light reflected from the surface of the colonies was quantified using the spot densitometry function of the Alpha-Imager (InnovTech). The light is reflected according to the degree of colony darkness: the darker colonies that produced more melanin reflect less light. For each strain at each incubation condition, at least 4 replicate spots were assayed. Each experiment was repeated three times.

### RNA extraction and RT-qPCR

The level of *LAC1* mRNA expression in the presence of nitrosative stress was tested using reverse transcriptase-quantitative PCR (RT-qPCR) in two representative strains, Y290-90 (clinical strain from Canada) and GRAKI13HO1-1 (environmental strain from Greece). These two strains were chosen because they had different melanin levels and showed different responses to nitrosative stresses. For each strain, cells were grown on caffeic acid medium with low, intermediate and high nitrosative stress as described above. After 4-days’ growth, cells were scraped off the solid medium and suspended in 1 mL sterile water and pelleted by centrifugation at 13,000 g for 2 minutes. The cell pellets were frozen in −80 °C and lyophilized overnight. The lyophilized pellets were resuspended in TRIzol Reagent (Invitrogen) and vortexed with glass beads (<1 mm) for 5 minutes. Total RNA was then extracted using TRIzol according to manufacturer’s instructions. Quantification of *LAC1* mRNA in the samples was achieved through RT-qPCR using Luna Universal One-Step RT-qPCR kit (NEB). *LAC1* primers spanning exon-exon junctions were used in qPCR to ensure exclusive amplification of mRNA. Actin 1, a single-copy housekeeping gene, was used as the reference to calibrate the relative expression levels between strains and among treatments for comparison. qPCR primers are listed in Table S1.

### Multilocus sequence typing (MLST) analysis

MLST was performed on all 54 strains according to the ISHAM consensus scheme^26^ and all novel sequences were deposited in the *Cryptococcus neoformans* MLST database. Data for the strains GRLMM26HO1-2, ITMPV22BK7-1, IUM 01-4729, IUM 01-4730, IUM 97-4899, IUM 98-2742, IUM 98-5036, NIH-430, PD32, TRNCGB1H01-1, NIH-433, NIH-424, IUM 02-0826, IUM 02-4295, IUM 93-1656, M9112, GRACA18BK1-3, GRACP15SO1-2, GRACP30BK1-1, GRAKI13HO1-1, GRAKI28HO1-1, IUM 01-0956, IUM 77-0033, IUM 79-0801, NIH-116, RKI 13-0490, RKI 13-0491, RKI 13-0492, PD1596, IUM 98-4987 were obtained from a previous study^33^.

### Sequencing of the *LAC1* gene

To obtain the nucleotide sequence of the *LAC1* gene (2886 bps) and its promoter (up to 2240 bps upstream of the transcription start site), we designed 6 primer pairs based on the fully sequenced genome of JEC21, available on NCBI (Accession number NC_006692.1, range 353455 to 357328 - reverse complement). Primers were designed to ensure sufficient overlap between the resulting fragments for accurate alignment and assembly. The primer sequences and their annealing temperatures are listed in Table S1 in Supplementary Information. Each fragment was PCR amplified and sequenced using Sanger method. All *LAC1* sequences were deposited into NCBI GenBank and their accession numbers are listed in Table 2. The software CLC Sequence Viewer 7.8.1 was used to combine the fragments to obtain the complete *LAC1* sequence for each strain. The “adegenet” package^36^ in R-studio was used to extract polymorphic sites from the aligned *LAC1* sequences of the population. Only those polymorphisms that were present at a Minor Allele Frequency (MAF) of or greater than 10% were included in downstream statistical analyses.

### Statistical Analysis

All statistical analyses were carried out using the R-Studio software and associated packages. One-way Analysis of Variance (ANOVA) was conducted to compare the average melanin production of the population between the 8 tested environmental conditions. Within each condition, student’s t-tests were conducted to compare average melanin production by source of isolation (clinical vs. environmental) and mating type (*MAT*a vs. *MAT*α). Within the conditions where the average melanin production was determined to be significantly different between the two mating types, one-way ANOVA was used to determine the percentage of melanin variance explained by the *MAT* locus. To partition melanin variance within each of the 3 types of stress (thermal, oxidative and nitrosative stress) into genotype effects, environmental effects and genotype-environment interaction effects, we conducted two-way ANOVAs with the first factor being the strain (we assumed each strain to be genetically unique from the rest of the population) and second factor being different levels of the stress. For each of the 8 tested conditions (i.e. different levels of thermal stress, oxidative stress and nitrosative stress), we used one-way ANOVA to determine if average melanin production significantly differed between *LAC1* haplotypes and to estimate the percentage of population’s melanin variance explained by the *LAC1* locus.

### Data availability

All data described in the study are presented in the manuscript. The new DNA sequences obtained and described in this study have been deposited in the *Cryptococcus neoformans* multilocus sequence typing database (http://mlst.mycologylab.org) and GenBank (accession numbers MG963844 to MG963895, Table 2).

### Statements on study approvals

We confirm that all methods in this study were carried out in accordance with relevant guidelines and regulations at McMaster University. In addition, all experimental protocols were approved by the President’s Biosafety Advisory Committee at McMaster University.

## Electronic supplementary material

Supplementary information
