# Author Correction: Genetic Factors and Genotype-Environment Interactions Contribute to Variation in Melanin Production in the Fungal Pathogen *Cryptococcus neoformans*

**DOI:** 10.1038/s41598-021-91476-w

**Published:** 2021-06-14

**Authors:** Himeshi Samarasinghe, David Aceituno-Caicedo, Massimo Cogliati, Kyung J. Kwon-Chung, Volker Rickerts, Aristea Velegraki, Sevim Akcaglar, Jianping Xu

**Affiliations:** 1 Department of Biology, McMaster University, 1280 Main Street West, Hamilton, ON Canada; 2 Department of Biomedical Sciences for Health, Università degli Studi di Milano, Milan, Italy; 3 Molecular Microbiology Section, Laboratory of Clinical Immunology and Microbiology, National Institute of Allergy and Infectious Diseases, NIH, Bethesda, MD USA; 4 Robert Koch Institute, Berlin, Germany; 5 Medical School National and Kapodistrian University of Athens, Athens, Greece; 6 School of Medicine, Uludag University, Bursa, Turkey

Correction to: *Scientific Reports* 10.1038/s41598-018-27813-3, published online 29 June 2018

The original version of this Article contained an error in the spelling of the author Sevim Akcaglar which was incorrectly given as Sevim Ackaglar. The original Article and accompanying Supplementary Information file have been corrected.

