# Supplementary material for: Genetic Factors and Genotype-Environment Interactions Contribute to Variation in Melanin Production in the Fungal Pathogen Cryptococcus neoformans
Source: Sci Rep. 2018 Jun 29;8:9824. doi: 10.1038/s41598-018-27813-3 (PMC6026209; doi:10.1038/s41598-018-27813-3)
Supplement: Supplementary file 1 — Supplementary information [file 41598_2018_27813_MOESM1_ESM.pdf]

Genetic Factors and Genotype-Environment Interactions Contribute to Variation in Melanin Production in the Fungal Pathogen *Cryptococcus neoformans*

Himeshi Samarasinghe[1], David Aceituno-Caicedo[1], Massimo Cogliati[2], Kyung J. Kwon-Chung[3], Volker Rickerts[4], Aristea Velegraki[5], Sevim Ackaglar[6], Jianping Xu*[1]

[1]Department of Biology, McMaster University, 1280 Main Street West, Hamilton, Ontario, Canada

[2]Department of Biomedical Sciences for Health, Università degli Studi di Milano, Milano, Italy

[3]Molecular Microbiology Section, Laboratory of Clinical Immunology and Microbiology, National Institute of Allergy and Infectious Diseases, NIH, Bethesda, Maryland, United States

[4]Robert Koch Institute, Berlin, Germany

[5]Medical School National and Kapodistrian University of Athens, Athens, Greece

[6]Uludag University, School of Medicine, Bursa, Turkey

*Corresponding author: Jianping Xu, Department of Biology, McMaster University, 1280 Main Street West, Hamilton, Ontario, L8S 4K1, Canada, email: jpxu@mcmaster.ca

Supplementary Table S1: Primers used to amplify the *LAC1* gene and its promoter from genomic DNA of *C. neoformans* var. *neoformans.* The primers used in RT-qPCR are also included here.

Primer name	Sequence (5' to 3')	Fragment length (bp)
LAC1P_F	TTGGGGCGCTGTAAACTAAC	1187
LAC1P_R	GGACAAAGGATGCTGGTGTT	
5UTR_F	TGACCAGCGAAACGAAGGAA	1117
5UTR_R	ACTGTTACCGGTGAGAAGTCG	
LCR1000_F	AACCATGCCAGTCTGGATTT	980
LCR1000_R	TCAACCCTTATCGGCACTTC	
LCR1.6_F	GGAGTGGCTAGAGCTGCAAT	898
LCR1.6_R	GTCGTTGAGTTTCGCAAGGT	
LCR2000_F	CATTCACAAAGGCTCCTCGT	1192
LCR2000_R	GGAATTGGTCTTAACTGATAAATTGG	
LCR2.6_F	TTCTCGTACCAAGCCGCATT	1008
LCR2.6_R	ACTCTTTCGTGAGCATTGGTTG	
LAC1_qPCR_F	GGTGATGCGTTTTGGCTGAG	147
LAC1_qPCR_R	CCGCTAGATCACTCCAAGCA	
Actin1_F	CTGCCCAAGTAAAACTCGGC	88
Actin1_R	CCAGCCTTGCACATACCAGA	
